# Purely endoscopic biportal and monoportal removal of the choroid plexus papilloma of the third ventricle with bilateral spread to the lateral ventricles

**DOI:** 10.3171/2023.1.FOCVID22170

**Published:** 2023-04-01

**Authors:** Albert A. Sufianov, Ilshat A. Gaysin, Iurii A. Iakimov, Rinat A. Sufianov

**Affiliations:** 1 Department of Pediatric Neurosurgery of Federal Center of Neurosurgery, Federal Center of Neurosurgery of Ministry of Health of the Russian Federation, Tyumen;; 2 Department of Neurosurgery, I.M. Sechenov First Moscow State Medical University (Sechenov University), Moscow;; 3 Educational and Scientific Institute of Neurosurgery, Peoples’ Friendship University of Russia (RUDN University), Moscow, Russian Federation; and; 4 King Edward Medical University, Lahore, Pakistan

**Keywords:** choroid plexus papilloma, endoscopic, biportal, monoportal

## Abstract

Modern neuroendoscopy makes it possible to treat tumors of various localizations with a reduced risk of intra- and postoperative complications. In this video, the authors present biportal and monoportal techniques for the removal of the choroid plexus papilloma of the third ventricle with bilateral spread to the lateral ventricles in a 1-year-old boy. For this operation, they successfully used a new instrument for neuroendoscopy, LigaSure, specially designed for intra-abdominal surgery.

The video can be found here: https://stream.cadmore.media/r10.3171/2023.1.FOCVID22170

## Transcript

This video case report demonstrates the purely endoscopic removal of choroid plexus papilloma of the third ventricle with bilateral spread to the lateral ventricles.

### 0:30 Patient History.

A 1-year-old boy presented with developmental delay and hydrocephalic signs, with a gradual deterioration. The boy was born with the signs of intraventricular hemorrhage. Neuroimaging revealed a tumor of the third ventricle.

### 0:45 Preoperative Imaging.

Preoperative MRI showed a homogenous contrast-accumulating tumor completely occupying the third ventricle with bilateral spread to the lateral ventricles, with a larger part on the left side and a typical picture of a choroid plexus tumor. Hydrocephalus with Evans index of 0.46 was also observed.

### 1:05 Operative Plan.

The first line of treatment for all choroid plexus tumors is maximal surgical resection.^1,2^ In this case, a two-stage operation was planned.

At the first stage, we performed a biportal removal of the tumor using transcortical intraventricular approach with trajectories planned on Brainlab neuronavigation system.^3–5^ The endoscope with neuroendoscopy instruments was inserted through one port, and additional instruments were inserted through the second port with a trajectory to the vascular pedicle for the safe tumor resection.

The second stage included monoportal removal of the remaining part of the tumor using a standard neuroendoscopic equipment.

### 1:50 Operative Video: First Stage.

The endoscope was guided through the port along the first trajectory directly to the part of the tumor in the left lateral ventricle. The initial viewing showed the main anatomical structures with the tumor arising from the choroid plexus near the foramen of Monro. The second instrument was inserted through the port under the visual control along the second trajectory.

In this case, we used LigaSure [Covidien, Medtronic] for neuroendoscopic tumor removal. This instrument was specially designed for intra-abdominal surgery. LigaSure has wide coagulating surface on the jaws. The cutting knife passes along the midline of the coagulation zone. In addition, rotation around the axis allows precise coagulation at different angles and directions.

The tumor was collected for pathology study. During the dissection, the instruments of the neuroendoscope helped to perform safe coagulation. The tumor was removed piece by piece. With the biportal approach, it was possible to change the trajectory of the main tool, by changing places of the tools in the ports. For bleeding control, the intraventricular manipulations were performed not only in the liquid environment but also in the dry field. The revision did not reveal the residual part of the tumor on the left side.

### 4:10 Operative Video: Second Stage.

The endoscope was guided directly toward the residual tumor, and we could visualize the main surrounding anatomical structures. The dissection was performed using standard endoscopic equipment, scissors, and a bipolar coagulator. The revision did not reveal any residual part of the tumor in the right lateral ventricle or the third ventricle.

### 5:06 Postoperative Imaging.

Early postoperative MRI proved gross-total resection of the tumor.

### 5:12 Postoperative Course.

In the early postoperative period, the patient had a mild transient right upper limb monoparesis. The follow-up examination of the patient after 4 years showed no recurrence.

## Disclosures

The authors report no conflict of interest concerning the materials or methods used in this study or the findings specified in this publication.

## Author Contributions

Primary surgeon: AA Sufianov. Assistant surgeon: Iakimov, RA Sufianov. Editing and drafting the video and abstract: all authors. Critically revising the work: AA Sufianov, Gaysin, RA Sufianov. Reviewed submitted version of the work: all authors. Approved the final version of the work on behalf of all authors: AA Sufianov. Supervision: AA Sufianov.

## Supplemental Information

### Patient Informed Consent

The necessary patient informed consent was obtained in this study.
